# Oncological and Reproductive Outcomes After Fertility-Sparing Surgery for Stage I Mucinous Ovarian Carcinoma

**DOI:** 10.3389/fonc.2022.856818

**Published:** 2022-07-04

**Authors:** Wei Lin, Dongyan Cao, Xiaohua Shi, Yan You, Jiaxin Yang, Keng Shen

**Affiliations:** ^1^ Department of Obstetrics and Gynecology, National Clinical Research Centre for Obstetrics and Gynecologic Diseases, Peking Union Medical College Hospital, Chinese Academy of Medical Sciences and Peking Union Medical College, Beijing, China; ^2^ Department of Pathology, Peking Union Medical College Hospital, Chinese Academy of Medical Sciences and Peking Union Medical College, Beijing, China

**Keywords:** epithelial ovarian cancer (EOC), mucinous carcinoma, fertility-sparing surgery (FSS), oncological outcome, reproductive outcome

## Abstract

**Background:**

Fertility-sparing surgery (FSS) has been widely used for patients with early-stage mucinous ovarian carcinoma (MOC). However, there is limited evidence regarding the reproductive outcomes as well as the impact of growth pattern on oncological outcomes after FSS. This study aims to evaluate the oncological and reproductive outcomes of patients with stage I primary MOC after FSS.

**Methods:**

This retrospective study enrolled 159 women with histologically confirmed unilateral stage I MOC treated at Peking Union Medical College Hospital between 1997 and 2019. Sixty-seven cases were pathologically reviewed for the growth pattern. Seventy-eight patients had FSS, defined as conservation of the uterus and at least part of one ovary, while 81 underwent radical surgery (RS). Oncofertility outcomes were compared between the groups and clinicopathological factors associated with disease-free survival (DFS) were analyzed by univariate and multivariate analyses. Patients in the FSS group were contacted to collect data on reproductive outcomes.

**Results:**

Eighteen patients developed recurrent disease during a median follow-up of 69 months, including 12 in the FSS and six in the RS group. There was one death each in the FSS and RS groups. There was no significant difference in DFS between the groups. CA125 >35 U/ml, stage IC, and incomplete staging were correlated with worse DFS according to multivariate analysis (*P*=0.001; 0.020 (stage IC) and 0.004 (incomplete staging) respectively). There was no significant difference in DFS between patients with stage IA and stage IC1 in the FSS group, while DFS was poorer in patients with stage IC2/3 than stage IA (*P*=0.028). In addition, DFS was significantly poorer in patients who underwent unilateral salpingo-oophorectomy (USO) compared with those receiving USO plus staging surgery (*P*=0.015). There was a tendency towards poorer DFS in the infiltrative tumors compared with the expansile tumors (*P*=0.056). Of 23 patients who attempted to conceive, 21 (91.3%) achieved 27 pregnancies, including 26 spontaneous pregnancies and one following assisted reproductive technology. Twenty patients gave birth to 24 healthy babies, including 21 full-term and three premature births. The live-birth rate was 88.9%.

**Conclusions:**

FSS is a suitable option for young women with unilateral stage I expansile MOC, with acceptable oncological outcomes and meaningful pregnancy rates. Re-staging should be proposed in patients who undergo incomplete staging surgery.

## Introduction

Epithelial ovarian cancer (EOC) is one of the most common cancers with a high mortality rate. In China, 52100 new cases are diagnosed and 22500 deaths are reported annually (1). Historically, hysterectomy and bilateral salpingo-oophorectomy (BSO) have been considered part of the standard surgical treatment of EOC (2). Approximately 10%-12% of EOC arise in patients younger than 40 years of age (3). Among these women, standard treatment causes permanent sterility (4), and thus negatively affects their quality of life (5). It is reasonable to consider fertility-sparing surgery (FSS) to maximize reproductive capacity without sacrificing treatment outcomes (6). Data on the use of FSS for early EOC have suggested that conservative treatment does not lead to an increase in mortality, poorer overall survival (OS), or shorter disease-free survival (DFS) (7–9). Current guidelines thus recommend FSS in selected patients with early-stage EOC who wish to preserve fertility (10, 11).

EOC is a heterogeneous tumor with diverse histological subtypes, each associated with unique clinicopathological and epidemiological characteristics (12), of which mucinous ovarian carcinoma (MOC) accounts for approximately 2%–3% of all EOC cases (6, 13). Most MOCs are unilateral and identified at an early stage, with an excellent prognosis (14). Patients typically present at a younger age than those with high-grade serous ovarian cancer (15), and patients with early-stage MOC are thus appropriate candidates for FSS (16, 17). Previous reports mainly focused on the safety of FSS in MOC, and few studies investigated the reproductive outcomes of patients after FSS (18–21). In 2014, World Health Organization (WHO) categorized MOC into two subtypes according to the growth pattern: the expansile and the infiltrative subtype (22). The expansile tumors suggest a lower metastatic potential, while the infiltrative tumors are more aggressive. There is little evidence about the impact of growth pattern on the oncological outcomes after FSS.

We conducted a single-center retrospective study to analyze the oncologic and reproductive outcomes of patients with stage I MOC who received FSS.

## Material and Methods

### Study Population

This retrospective cohort study was carried out using data from the computerized database at Peking Union Medical College Hospital (PUMCH). One hundred and fifty-nine consecutive patients with stage I MOC who were treated between January 1997 and September 2019 were included in this study. At the time of diagnosis, the histological slides of all patients were reviewed in our center, including referral patients. When this retrospective study was conducted, the original slides of some referral cases were unavailable, and some slides were too old to provide sufficient tissues to make a definitive diagnosis for the growth pattern. Finally, a total of 67 cases were reviewed for the growth pattern by two dedicated gynecologic pathologists according to the 2014 WHO criteria. The exclusion criteria were as follows: (1) patients with metastatic MOC from a primary extraovarian tumor; (2) patients with a borderline mucinous tumor, intraepithelial carcinoma, or stromal invasion limited to an area ≤10 mm^2^; (3) patients with bilateral tumors; and (4) patients with concurrent malignancies in another site. The methods used to distinguish between primary and metastatic MOC have been described previously (23). The study was approved by the Institutional Review Board (IRB) of PUMCH (S-K1802) in accordance with the Declaration of Helsinki. Verbal informed consent was obtained during follow-up visits or telephone interviews.

Patient data were obtained from inpatient and outpatient records, including demographics, clinical features, surgical procedures, pathological findings, adjuvant chemotherapy, treatment after relapse, and follow-up information. Telephone interviews with patients and their family members were conducted to obtain additional information on menstruation and reproductive outcomes. In accordance with the journal’s guidelines, we will provide our data for the reproducibility of this study in other centers if such is requested.

### Treatment and Follow-Up

FSS, defined as conservation of the uterus and at least part of one ovary, included unilateral cystectomy (UC) and unilateral salpingo-oophorectomy (USO). Radical surgery (RS) was defined as bilateral salpingo-oophorectomy or hysterectomy (with unilateral or bilateral salpingo-oophorectomy). All patients were categorized into the FSS or RS group based on their initial surgery.

Complete surgical staging included exploration of the entire peritoneal cavity, peritoneal cytology, omentectomy or omental biopsy, multiple peritoneal biopsies, and biopsy of any suspicious area. Surgical staging was considered incomplete in all other cases, regardless of the conservative or radical nature of the surgery. Appendectomy and lymphadenectomy were optional and carried out according to the surgeons’ experience and intraoperative findings. Appendectomy was performed in 150 (94.3%) patients. Among them, 16 (10.7%) appendices were grossly abnormal during surgery but none was found microscopic involvement. Two patients were surgical staged but didn’t undergo appendectomy. One hundred and thirty-five (84.9%) patients underwent lymphadenectomy. The rate of lymphadenectomy had declined over time, from nearly 90% before 2004 to 80% after 2014.

Disease stage was determined according to the International Federation of Gynecology and Obstetrics (FIGO) criteria (2014) (24). Stage IC tumors were further classified as IC1 (intraoperative capsule rupture), IC2 (preoperative capsule rupture or tumor on ovarian surface), and IC3 (malignant cells in ascites or peritoneal washings). Patients with insufficient data to classify them with IC disease were classified as having ICX disease. Following surgery, patients were treated with adjuvant platinum-based chemotherapeutic regimens in accordance with National Comprehensive Cancer Network guidelines (11, 25–28) or based on surgeons’ experience (for some old cases).

After initial treatment, follow-up was scheduled every 3 months during the first year, every 6 months during between the second and fifth years, and annually thereafter. Surveillance included symptom review, physical and pelvic examinations, measurement of serum tumor markers, and periodic imaging. The last follow-up appointment was on August 26, 2021. DFS was defined as the time interval from the date of diagnosis to the date of recurrence or censoring. OS was calculated as the time interval from the date of diagnosis to the date of death or censoring. Patients who were lost to follow-up within 6 months after the initial surgery or with insufficient clinical data were excluded from the survival analysis. There was no significant difference in the demographic and clinical characteristics of patients between the included cohort and the excluded cohort (**Supplementary Table 1**).

### Statistical Analysis

Continuous variables were compared using Student’s *t-*test or Mann–Whitney U test. Frequencies of categorical variables were compared using Pearson χ^2^ or Fisher’s exact test. Survival curves and rates were calculated using the Kaplan–Meier method. Differences in survival between groups were compared using the log-rank test in univariate analysis. Multivariate analysis was performed using the Cox proportional hazards model. The results were expressed as hazard ratios and 95% confidence intervals. A two-sided *P* value <0.05 was considered significant. All statistical analyses were performed using SPSS version 26.0 (IBM Corporation, Armonk, NY, USA).

## Results

### Patient Characteristics

A total of 159 patients with unilateral stage I MOC were treated in or referred to our institution between 1997 and 2019. Sixty-seven patients were reviewed for the growth pattern, including 55 expansile and 12 infiltrative subtypes. Seventy-eight (49.1%) patients underwent FSS. The rates of FSS were 14.3% for women diagnosed between 1997 and 2000 and 65.1% for those diagnosed between 2016 and 2019.

The median age of patients in the FSS group was 24 years. Intra-operative frozen section was performed in 49 (62.8%) patients. Among them, 34, 13 and 2 were reported as malignant, borderline and benign tumors, respectively. FIGO stages IA, IC1, IC2, and IC3 were noted in 29 (37.2%), 31 (39.7%), 15 (19.2%), and two (2.6%) patients, respectively. Thirty-two (41.0%) patients experienced intra-operative rupture. Among them, 28 patients underwent primary surgery in other institutions and 4 patients in our institution. Platinum-based adjuvant chemotherapy was administered to 44 (56.4%) patients, including 11 IA patients and 33 IC patients. The proportion of IC patients was significantly higher in the chemotherapy group than in the non-chemotherapy group (75.0% vs 47.1%, *P*=0.011).

**Table 1** summarizes the baseline characteristics of patients stratified by surgery type. There was no significant difference between the FSS group and RS group regarding preoperative CA125 value, frequency of complete staging surgery, distribution of FIGO stage, rate of chemotherapy, and distribution of growth pattern.

**Table 1:** Demographic and clinical characteristics of patients.

Characteristic	Total (N=159)	FSS (N=78)	RS (N=81)	*P* value
Median age, years (range)	31 (12-76)	24 (12-40)	45 (22-76)	<0.001
Nulliparous	79 (50.3%)	65 (83.3%)	14 (17.7%)	<0.001
Elevated serum CA125^a^	52 (36.9%)	24 (33.8%)	28 (40.0%)	0.446
Median tumor diameter, cm (range)^b^	15 (3.9-40.0)	18.0 (5.0-40.0)	15.0 (3.9-30.0)	0.020
Complete staging	147 (92.5%)	69 (88.5%)	78 (96.3%)	0.062
FIGO stage				0.501
IA	55 (34.6%)	29 (37.2%)	26 (32.1%)	
IC	104 (65.4%)	49 (62.8%)	55 (67.9%)	
Substage				
IC1	63 (39.6%)	31 (39.7%)	32 (39.5%)	
IC2	33 (20.8%)	15 (19.2%)	18 (22.2%)	
IC3	4 (2.5%)	2 (2.6%)	2 (2.5%)	
ICX	4 (2.5%)	1 (1.3%)	3 (3.7%)	
Chemotherapy	100 (62.9%)	44 (56.4%)	56 (69.1%)	0.097
Growth pattern^c^				0.512
Expansile	55	24	31	
Infiltrative	12	4	8	

FSS, fertility-sparing surgery; RS, radical surgery.

a Data are available in 141 patients.

b Data are available in 147 patients.

c Data are available in 67 patients.

**Figure 1:**
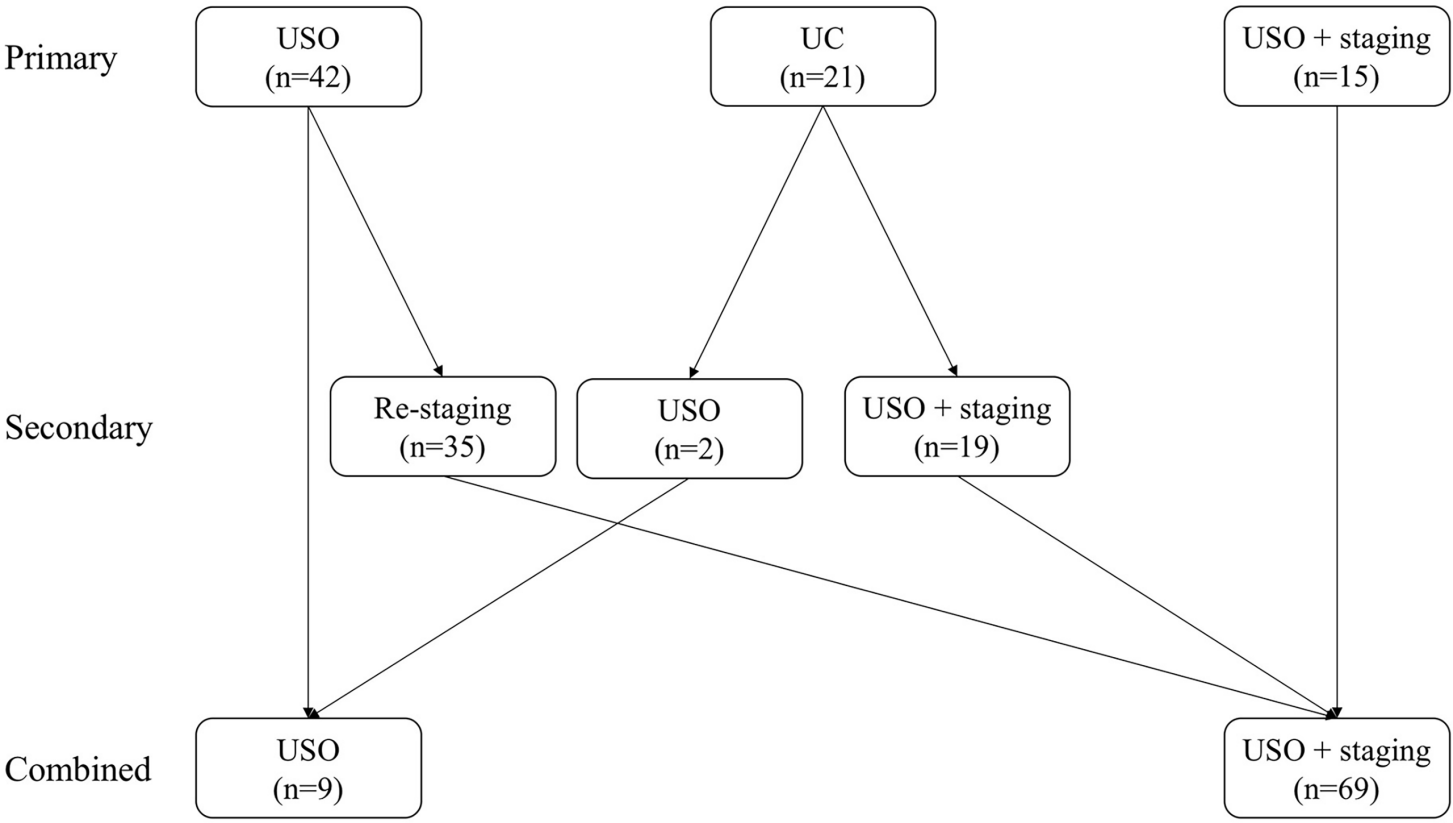
Initial surgical procedures in the FSS group. (USO, unilateral salpingo-oophorectomy; UC, unilateral cystectomy).

Details of the surgical procedures in the FSS group are shown in **Figure 1** and **Table 2**. Patients were divided into three subgroups based on their primary surgery: USO, UC, and USO + staging. Thirty-five patients (83.3%) in the USO group underwent restaging secondary surgery, and all patients (n=21) in the UC group underwent secondary surgery (USO, n=2; USO + staging, n=19). Of these 21 patients, four were found to have remaining tumor lesions in the preserved ovary. After combining the extents of the primary and secondary surgeries, there were nine patients in the USO group and 69 patients in the USO + staging group. When it comes to minimally invasive surgery, 30 (38.5%) patients received at least one laparoscopy. Among them, 9 patients underwent laparotomic USO or UC first, and then laparoscopic re-staging surgery. Eleven patients had USO or UC *via* laparoscopy in the primary surgery, and surgical staging *via* laparotomy later. No node metastases were found in the 64 patients who underwent retroperitoneal lymphadenectomy.

**Table 2:** Characteristics of initial surgery in the FSS group (n=78).

Characteristic	N
Surgical approach	
Laparoscopy	10 (12.8%)
Laparotomy	48 (61.5%)
Combined modality	20 (25.6%)
Staging procedures	
Peritoneal biopsy or cytology	65 (85.5%)
Omentectomy	69 (88.5%)
Appendectomy	73 (93.6%)
Lymphadenectomy	64 (82.1%)
Median number of nodes removed (range)	23 (1-73)
Contralateral ovary biopsy	40 (51.3%)
Staging surgery	
One-step	15 (19.2%)
Two-step	54 (69.2%)
No (only USO)	9 (11.5%)

FSS, fertility-sparing surgery; USO, unilateral salpingo-oophorectomy.

### Oncological Outcomes

The median follow-up interval for all patients was 69 months (range, 6–240 months). Eighteen (11.3%) patients developed at least one recurrence, including 12 (15.4%) patients in the FSS group and six (7.4%) in the RS group (*P*=0.112). The median times to recurrence were 15 months (range, 5–99 months) for all patients, 14 months for the FSS group, and 28 months for the RS group (*P*=0.206). There was one (1.2%) death in the RS group and one (1.3%) in the FSS group. The 5-year DFS rates for the whole cohort, FSS group, and RS group were 88.6%, 82.5%, and 94.5% (*P*=0.207), respectively, and the corresponding 5-year OS rates were 99.3%, 98.6% and 100% (*P*=0.916), respectively.

Detailed information on the 12 relapsed patients in the FSS group is summarized in **Table 3**. Tumor stage was IA in one patient and IC in 11 patients. The recurrence sites included the contralateral ovary (9 patients), peritoneum (5 patients), abdominal wall (3 patients), diaphragm (3 patients), pelvic lymph nodes (3 patients), omentum (2 patients), and other sites. After relapse, ten (83.3%) patients received both surgery and chemotherapy, one (8.3%) patient with distant recurrence was treated with chemotherapy alone, and another patient only underwent surgery. At the last follow-up, eight patients were alive with disease and three patients remained alive with no evidence of disease. One patient developed recurrence at multiple sites with no response to chemotherapy and died of MOC. Growth pattern data were available in 4 patients. Two patients with expansile tumors were alive with no evidence of disease, while another two with infiltrative tumors were alive with disease.

**Table 3:** Clinical details of the 12 relapsing patients in the FSS group.

No.	Age	Stage	Initial treatment		DFS	Recurrence			OS	Status
			Surgery	Chemo		Contralateral ovary	Other sites	Treatment		
1	32	IA	USO + re-staging	No	43	Yes	Peritoneum, diaphragm	CRS + Chemo	73	AWD
2^a^	22	IC1	USO + staging	No	36	No	Lung, mediastinal LN, thoracic vertebra	Chemo	46	AWD
3	18	IC1	USO	Yes	5	Yes	Peritoneum, omentum	CRS + Chemo	16	AWD
4	23	IC1	USO	Yes	8	No	Abdominal wall, pelvic LN	CRS + Chemo	21	AWD
5^b^	31	IC1	USO + re-staging	No	34	Yes	/	CRS	69	NED
6	23	IC1	USO + staging	No	12	Yes	Pleural effusions, peritoneum, omentum, pelvic LN	CRS + Chemo	15	DOD
7	27	IC1	USO + staging	Yes	15	Yes	/	TAH + USO + Chemo	24	AWD
8^a^	37	IC2	USO + staging	Yes	13	Yes	Peritoneum, abdominal wall, ileum	CRS + Chemo	16	AWD
9	29	IC2	USO	Yes	8	No	Inguinal LN	LND + Chemo	21	AWD
10^b^	24	IC3	USO + staging	Yes	16	Yes	Peritoneum	CRS + Chemo	39	NED
11	26	IC3	USO + staging	Yes	41	Yes	Diaphragm	CRS + Chemo	61	NED
12	25	ICX	USO + staging	Yes	7	Yes	Diaphragm, abdominal wall, pelvic LN	CRS + Chemo	27	AWD

AWD, alive with disease; Chemo, chemotherapy; CRS, cytoreductive surgery; DFS, disease-free survival; DOD, died of disease; FSS, fertility-sparing surgery; LN, lymph node; LND, lymphadenectomy; NED, no evidence of disease; OS, overall survival; TAH, total abdominal hysterectomy; USO, unilateral salpingo-oophorectomy.

a No. 2 patient and No. 8 patient were diagnosed as infiltrative tumors.

b No. 5 patient and No. 10 patient were diagnosed as expansile tumors.

**Figure 2:**
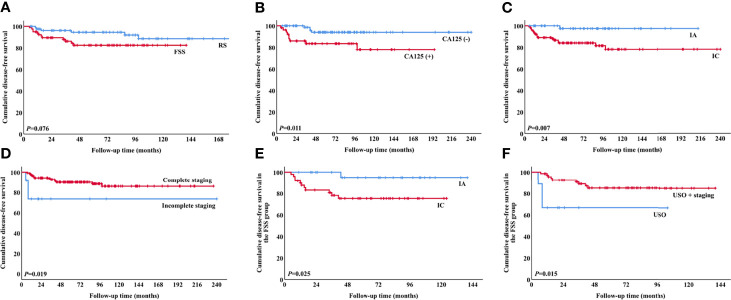
Survival curves for patients. DFS according to **(A)** surgical procedure (*P*=0.076), **(B)** preoperative CA125 values (*P*=0.011), **(C)** stage (*P*=0.007), **(D)** completeness of staging surgery (*P*=0.019), **(E)** stage in the FSS group (*P*=0.025), and **(F)** surgical procedure in the FSS group (*P*=0.015). (DFS, disease-free survival; FSS, fertility-sparing surgery).

Univariate analysis revealed a tendency towards poorer DFS in the FSS group compared with the RS group (**Figure 2A**), but the result was not significant (*P*=0.076). Multivariate analysis indicated that FSS had no substantial impact on DFS (**Table 4**). CA125 >35 U/ml, stage IC, and incomplete staging were correlated with worse DFS (**Figures 2B–D**). Following stratification by disease sub-stage, the 5-year DFS rate for patients with stage IA who underwent FSS (n=29) was 95.0%, compared with 100.0% (*P*=0.330) for those who underwent RS (n=26). For women with stage IC disease, the 5-year DFS rate in the FSS group (n=49) was 75.2% compared with 92.0% (*P*=0.056) in women who underwent RS (n=55). It was not possible to perform a multivariate analysis of OS because of the low number of death events.

**Table 4:** Univariate and multivariate analyses of prognostic factors related to DFS.

Prognostic factors	N	Univariate analysis		Multivariate analysis	
		5-year DFS (%)	*P* value	HR (95% CI)	*P* value
Surgical procedure			0.076		0.586
FSS	78	82.5		2.072 (0.151-28.416)	
RS	81	94.5		1	
Age (years)			0.016		0.488
≤40	111	85.2		3.120 (0.125-77.651)	
>40	48	97.0		1	
CA125 (U/mL)			0.011		0.001
≤35	89	94.0		1	
>35	52	83.5		12.318 (2.696-56.283)	
Tumor size (cm)			0.916		0.769
<10	25	91.6		1.292 (0.233-7.159)	
≥10	125	89.6		1	
FIGO stage			0.007		0.020
IA	55	97.4		1	
IC	104	84.1		18.566 (1.577-218.640)	
Complete staging			0.019		0.004
Yes	147	89.9		1	
No	12	73.3		38.126 (3.285-442.435)	
Adjuvant chemotherapy			0.457		0.282
Yes	100	87.2		0.396 (0.073-2.142)	
No	59	91.0		1	
Surgical approach			0.007		0.341
Laparotomy	136	91.7		1	
Laparoscopy	23	69.7		2.038 (0.471-8.825)	
Initial treatment			0.017		0.201
Our institution	77	94.2		1	
Other institutions	82	83.5		3.126 (0.546-17.907)	

CI, confidence interval; DFS, disease-free survival; FSS, fertility- sparing surgery; HR, hazard ratio; N, number; RS, radical surgery.

Regarding the 78 patients in the FSS group, stage IC was significantly associated with poor DFS (*P*=0.025, **Figure 2E**). There was no significant difference in DFS between patients with stage IA and those with stage IC1 (*P*=0.053), but DFS was significantly poorer in patients with stage IC2/3 compared with stage IA (*P*=0.028). The 5-year DFS rates of patients who underwent USO and USO + staging were 66.7% and 85.0%, respectively (*P*=0.015, **Figure 2F**). In the USO + staging subgroup, there was no significant difference in DFS between one-step and two-step surgery (*P*=0.389). For the 28 patients with growth pattern data, there was a tendency towards poorer DFS in the infiltrative tumors compared with the expansile tumors, although the result was not significant (*P*=0.056).

### Fertility Outcomes

Fifty-seven patients in the FSS group were contacted to collect information on reproductive outcomes. Of these, 34 patients (59.6%) were single or using contraceptives and 23 (40.4%) attempted to conceive after surgery. During a median follow-up of 73 months (range, 20–115 months), 21 patients (91.3%) achieved 27 pregnancies, including 26 spontaneous pregnancies and one pregnancy following assisted reproductive technology. Twenty patients gave birth to 24 healthy babies, including 21 full-term births (2 *via* vaginal delivery and 19 *via* cesarean section) and three premature births (all *via* cesarean section). The live-birth rate was 88.9%. The cesarean section rate was 91.7%, due to fetal distress, scarred uterus, maternal request and so on.

Among the 100 patients who received chemotherapy, only 14 (14%) patients desired to have babies after treatment. Of these, 13 patients achieved 17 spontaneous pregnancies. All patients but one gave birth to healthy babies. This patient had two fetal losses; the first pregnancy was terminated at 23 weeks due to oligohydramnios and fetal growth restriction, and her second pregnancy was terminated at 26 weeks due to early-onset preeclampsia and fetal growth restriction.

Among the 21 patients who achieved pregnancies, 2 (9.5%) cases developed recurrence. One patient delivered a baby 20 months after the completion of initial treatment, but recurred 18 months thereafter. She was treated with cytoreductive surgery and chemotherapy after relapse. Another patient succeed to conceive 12 months after the last cycle of adjuvant chemotherapy. She was diagnosed with recurrent disease during pregnancy, and received cesarean section and cytoreductive surgery at 34 weeks. At the last follow-up, these two patients were both alive with no evidence of disease.

## Discussion

This study investigated the safety and effectiveness of FSS in patients with stage I MOC treated at a tertiary center in China. We observed that patients who were treated conservatively showed an acceptable DFS compared with those who underwent RS. In addition, we noted some pregnancies and deliveries among patients in the FSS group, indicating a certain benefit of FSS in young patients.

The present cohort included consecutive patients with unilateral stage I MOC treated over a 20-year period. We observed a significant increase in the rate of FSS over the study period. Our results agree with the current body of literature that supports the safety of FSS among young patients with stage I MOC (18, 19, 21). Although patients treated with FSS had a higher recurrence rate and shorter median interval to recurrence than those treated with RS, the difference was not significant. In addition, three quarters of the relapses in the FSS group occurred in the residual ovary, and most relapsed patients were successfully salvaged by surgery and chemotherapy. Patients are therefore advised to try to conceive as soon as possible after finishing their initial treatment, and should be followed up closely and receive timely treatment in the event of relapse.

The selection criteria for FSS candidates remains controversial. In the present study, there was no significant difference in DFS between the FSS and RS groups in patients stratified to stage IA and stage IC. However, a multicenter study by Morice et al. (29) suggested that FSS should only be undertaken in EOC patients with stage IA, while others considered that stage IC patients with grade 1 or grade 2 were also eligible for FSS (2, 7, 30). The current study suggests that DFS was poorer in patients with stage IC2/3 compared with stage IA, consistent with the retrospective study by Kajiyama et al. (31). In light of the relatively small number of patients with stage IC2/3 in the current study, FSS may be considered after obtaining adequate informed consent, but further studies are warranted to confirm the safety of FSS in this subgroup of patients.

USO is usually proposed for young patients wishing to preserve their fertility (32). Occasionally, cystectomy is the only FSS choice for patients with a previous history of salpingo-oophorectomy. An Italian study of women who underwent FSS found a recurrence rate for cystectomy of 17% (2). Kajiyama et al. (33) retrospectively evaluated eight EOC patients treated with FSS by cystectomy, and recommended adding postoperative chemotherapy in these patients to prevent occult tumors. In the present study, 21 patients underwent UC as primary surgery, with the risk of an occult tumor remaining in the preserved ovary of 19%. We therefore suggest carrying out USO instead of simple cystectomy in patients with MOC, except in particular cases.

The current results indicated that incomplete staging was an independent prognostic factor of DFS in patients with MOC. A previous large randomized trial involving 448 patients with early-stage EOC found that complete staging was associated with significant improvements in overall and recurrence-free survival (34). Incomplete staging may omit occult metastatic disease and lead to undertreatment in patients with high risk factors. Young et al. reported an upstaging rate of 31% among patients with presumed clinical early-stage EOC (35). In our study, 175 patients (some of them were excluded from the present cohort) were macroscopic stage I, and 7 (4%) patients were upstaged after surgical staging. Among them, 2 patients had nodal involvement (stage IIIA), 2 had microscopic omental disease (stage IIIB), and 3 exhibited pelvic spread on the peritoneum (stage IIB). Complete surgical staging is thus important in patients who want to preserve fertility (36).

According to the 2014 WHO criteria, MOCs are classified as the expansile subtype and the infiltrative subtype (22). Gouy et al. (20) firstly compared the results of FSS in patients with expansile subtype and those with the infiltrative subtype, and concluded FSS could be safely used for both subtypes. Our results were consistent with the previous study, but we should not ignore that there was a tendency towards poorer DFS in the infiltrative subgroup. Since the case numbers of both two studies are small, large-cohort studies with growth pattern data are warranted to strengthen the power.

Regarding the obstetrical outcomes, the overall conception rate in the current cohort was favorable, and most of the observed pregnancies were spontaneous. However, information on this issue is currently scarce (20, 37). Pregnancy planning may also be delayed in patients who receive adjuvant chemotherapy, to decrease the risk of pregnancy complications such as oligohydramnios and preeclampsia (17, 38).

Lymphatic involvement in MOC grossly confined to the ovary is rare (39, 40). Recent studies proposed that pelvic and paraaortic lymphadenectomy should be performed in the infiltrative type regardless of stage, and can be omitted in stage I expansile MOC (32). In our cohort, 84.9% of patients underwent lymphadenectomy, including 46 expansile and 11 infiltrative subtypes. Lymph node metastasis was not found in any of them. Therefore, our results reinforce that routine lymphadenectomy may be of little value in identifying metastasis in stage I MOC. We suggest that further studies focus on the role of lymphadenectomy in the infiltrative subtype.

Over the past two decades, appendectomy seemed to be a routine procedure in MOC to rule out the possibility of an appendiceal tumor that may metastasize to the ovaries. Nowadays, studies show that routine removal of the appendix is not required if the appendix appears grossly normal (41, 42). In our study, appendectomy was performed in 150 patients. Among them, 89.3% of appendices were grossly normal and 10.7% were abnormal, but none was found microscopic involvement. Our results were consistent with prior studies.

There is no clear evidence that adjuvant chemotherapy is beneficial in early-stage MOC (34). Recent studies show that adjuvant chemotherapy was not associated with improved OS in patients with stage I MOC (43, 44). In the present cohort, 62.9% of patients received adjuvant chemotherapy, which was a relatively high rate. After patients were stratified to stage IA and stage IC, there was no difference in DFS between patients who did and did not receive adjuvant chemotherapy in each substage. Based on our data, adjuvant chemotherapy does not contribute to the improving DFS of stage I MOC.

Recent studies have shown the rates of concordance between frozen section and final pathology diagnosis of mucinous ovarian tumors varied from 66% to 97.2% (45, 46). The discordance might be explained by the large size of mucinous ovarian tumors, and the heterogeneous lesions within one tumor (benign, borderline, and invasive carcinomas) (47). Based on our data, the concordance rate was 69.4%, which supported the previous findings. Therefore, the accurate diagnosis of MOC based on intraoperative frozen section examination can be difficult. Surgeons may pay more attention to the solid component in the tumors.

This study had several limitations. First, it was a retrospective study conducted in a single referral center, and may thus have included selection biases. Second, the age and parity distributions differed between the FSS and RS groups. However, we used multivariate logistic regression to adjust for these possible confounding factors. Thirdly, the cohort size was relatively small, which may result in the trend toward worse DFS in the FSS group but without statistically significant difference. Finally, we did not investigate tumor grade. There is a consensus that the grading system used for serous cancers should not be used for mucinous subtypes (20). In a large retrospective study, Crafton et al. (16) observed that FSS was unrelated to survival in patients with EOC in subgroups defined by stage and grade, and another study by Fruscio et al. (2) demonstrated that FSS may be proposed safely for all women with stage I EOC, regardless of tumor grade.

The main strengths of the present study were the inclusion of all consecutive MOC patients, and the long follow-up duration. In addition to oncologic outcomes, we also focused on patients’ pregnancy outcomes, which have rarely been reported in relevant studies. In addition, we included a comparison group of patients who received standard RS to allow us to draw more definitive conclusions. Last but not least, our study is the second to distinguish survival between expansile and infiltrative subtypes regarding the role of FSS, and adds new evidence to this topic.

## Conclusions

In conclusion, FSS is feasible and safe in young patients with unilateral stage I MOC, with good obstetrical outcomes. Patients should be selected carefully, especially those with infiltrative subtype, and should be thoroughly informed, completely staged, and closely followed-up. There is no sufficient evidence on the benefit of completion surgery after patients’ childbearing. Ethical and practical problems in patient recruitment make randomized controlled trials difficult, and further well-designed multicenter investigations with long-term follow-up are warranted to extrapolate the findings of this retrospective analysis into clinical practice.

## Data Availability Statement

The raw data supporting the conclusions of this article will be made available by the authors, without undue reservation.

## Ethics Statement

The studies involving human participants were reviewed and approved by The Institutional Review Board of Peking Union Medical College Hospital. Written informed consent from the participants’ legal guardian/next of kin was not required to participate in this study in accordance with the national legislation and the institutional requirements.

## Author Contributions

WL contributed to the conception and design of the study, the acquisition, analysis and interpretation of data, and manuscript drafting. DC undertook the conception and design of the study, the analysis and interpretation of data, and manuscript revising. XS and YY carried out the acquisition, analysis and interpretation of data. JY and KS performed the analysis and interpretation of data, and manuscript revising. All authors read and approved the final manuscript.

## Funding

This study is supported by the Non-profit Central Research Institute Fund of Chinese Academy of Medical Sciences (NO. 2020-PT320-003).

## Conflict of Interest

The authors declare that the research was conducted in the absence of any commercial or financial relationships that could be construed as a potential conflict of interest.

## Publisher’s Note

All claims expressed in this article are solely those of the authors and do not necessarily represent those of their affiliated organizations, or those of the publisher, the editors and the reviewers. Any product that may be evaluated in this article, or claim that may be made by its manufacturer, is not guaranteed or endorsed by the publisher.

## Supplementary Material

Supplementary Table 1 Demographic and clinical characteristics of patients in two cohorts
