# Supplementary material for: Oncological and Reproductive Outcomes After Fertility-Sparing Surgery for Stage I Mucinous Ovarian Carcinoma
Source: Front Oncol. 2022 Jul 4;12:856818. doi: 10.3389/fonc.2022.856818 (PMC9289154; doi:10.3389/fonc.2022.856818)
Supplement: Supplementary Table 1 — Demographic and clinical characteristics of patients in two cohorts [file Table_1.pdf]

Supplementary Table 1. Demographic and clinical characteristics of patients in two cohorts

Characteristics	Included cohort (N=159)	Excluded cohort (N=17)	*P* value
Median age, years (range)	31 (12-76)	26 (17-67)	0.321
Nulliparous	79 (50.3%)	9 (52.9%)	0.837
Elevated serum CA125	52 (36.9%)	5 (38.5%)	0.565
Median tumor diameter, cm	15 (3.9-40.0)	15 (2.2-30.0)	0.508
Complete staging	147 (92.5%)	13 (76.5%)	0.053
FIGO stage			0.668
IA	55 (34.6%)	5 (29.4%)	
IC	104 (65.4%)	12 (70.6%)	
Substage			
IC1	63 (39.6%)	6 (50%)	
IC2	33 (20.8%)	2 (16.7%)	
IC3	4 (2.5%)	0	
ICX	4 (2.5%)	4 (33.3%)	
